# The antibacterial effect of the combination of red raspberry concentrate and antibiotics on Staphylococcus aureus

**DOI:** 10.1186/1753-6561-9-S7-A1

**Published:** 2015-10-27

**Authors:** Aleksandar Bokan, Saša Vukmirović, Deana Medić

**Affiliations:** 1 University of Novi Sad, Faculty of Medicine Novi Sad, Hajduk Veljkova 3, Novi Sad 21000, Republic of Serbia; 2 Institute of pharmacology, Toxicology and Clinic Pharmacology, Hajduk Veljkova 3, Novi Sad 21000, Republic of Serbia; 3 Institute of Public Health of Vojvodina, Center for Microbiology, Futoska 121, Novi Sad 21000, Republic of Serbia

## Background

The increasing presence of both methicillin-resistant isolates of Staphylococcus aureus (MRSA), and lately vancomycin-resistant Staphylococcus aureus (VRSA), requires pursuit of new antibiotics. This is achieved in several ways: by modification of existing antibiotics, by synthesis of new antibiotics or by empirical screening of, so far, unexamined compounds. Consumption of fresh or processed berries (raspberry, blackberry, strawberry, currant, cranberry and other berries) is considered exceptionally beneficial due to the potent antioxidant and antibacterial activity of their phenolic compounds. Having in mind high percentage of highly resistant bacterial strains of Staphylococcus aureus and antibacterial potency of red raspberries' phenolic compounds, the goal of study was to investigate interactive effect of red raspberries' concentrate and antibiotics on Staphylococcus aureus [1-4].

## Methods

In the experiment we used: 1) absolute physicochemically unchanged, 100% natural raspberry juice, 2) clinical isolate of Staphylococcus aureus, 3) antibiotic discs for examination of bacterial susceptibility, 4) sterile 0.9% sodium-chloride and 5) Müller-Hinton agar. Testing the existence and type of interaction between the red raspberries' concentrate and the antimicrobial drugs was performed in vitro using Kirby-Bauer disc diffusion method in accordance with CLSI guidelines.

## Results

The results of our study clearly indicate the existence of synergism between penicillin, cefoxitin, tetracycline, ciprofloxacin and fusidic acid with red raspberries' concentrate as the source of various phenolic compounds.

## Conclusions

New classes of antibiotics are extremely necessary and flavonoids represent a potentially new group of antimicrobial substances that could potentiate the activity of conventional antibiotics.
